# Global prevalence of cardiometabolic risk factors in the military population: a systematic review and meta-analysis

**DOI:** 10.1186/s12902-020-0489-6

**Published:** 2020-01-13

**Authors:** Fereshteh Baygi, Kimmo Herttua, Olaf Chresten Jensen, Shirin Djalalinia, Armita Mahdavi Ghorabi, Hamid Asayesh, Mostafa Qorbani

**Affiliations:** 1 Centre of Maritime Health and Society, Institute of Public Health, University of Southern Denmark, Esbjerg, Denmark; 2 Non-communicable Diseases Research Center, Endocrinology and Metabolism Population Sciences Institute, Tehran University of Medical Sciences, Tehran, Iran; 3 Deputy of Research and Technology, Ministry of Health and Medical Education, Tehran, Iran; 4 Department of Medical emergency, Qom University of Medical Sciences, Qom, Iran; 5 Non-communicable Diseases Research Center, Alborz University of Medical Sciences, Karaj, Iran; 6 Endocrinology and Metabolism Research Center, Endocrinology and Metabolism Clinical Sciences Institute, Tehran University of Medical Sciences, Tehran, Iran

**Keywords:** Metabolic syndrome, Obesity, Military personnel, Systematic review

## Abstract

**Background:**

Although there are numerous studies on the global prevalence of cardiometabolic risk factors (CMRFs) in military personnel, the pooled prevalence of CMRFs in this population remains unclear. We aimed to systematically review the literature on the estimation of the global prevalence of CMRFs in the military population.

**Methods:**

We simultaneously searched PubMed and NLM Gateway (for MEDLINE), Institute of Scientific Information (ISI), and SCOPUS with using standard keywords. All papers published up to March 2018 were reviewed. Two independent reviewers assessed papers and extracted the data. Chi-square-based Q test was used to assess the heterogeneity of reported prevalence among studies. The overall prevalence of all CMRFs, including overweight, obesity, high low-density lipoprotein (LDL), high total cholesterol (TC), high triglyceride (TG), low high-density lipoprotein (HDL), hypertension (HTN) and high fasting blood sugar (FBS) was estimated by using the random effects meta-analysis. A total of 37 studies met the eligibility criteria and were included in the meta-analysis.

**Results:**

According the random effect meta-analysis, the global pooled prevalence (95% confidence interval) of MetS, high LDL, high TC, high TG, low HDL and high FBS were 21% (17–25), 32% (27–36), 34% (10–57), 24% (16–31), 28% (17–38) and 9% (5–12), respectively. Moreover, global pooled prevalence of overweight, generalized obesity, abdominal obesity and HTN were estimated to be 35% (31–39), 14% (13–16), 29% (20–39) and 26 (19–34), respectively.

**Conclusions:**

The overall prevalence of some cardio-metabolic risk factors was estimated to be higher in military personnel. Therefore, the necessary actions should be taken to reduce risk of developing cardiovascular diseases.

**Systematic review registration number in PROSPERO:**

CRD42018103345

## Key messages

- The global prevalence of metabolic syndrome in the military population was estimated to be 21%.
- The overall prevalence of obesity in the military population was estimated to be 14%.
- There was considerable variation in the overall prevalence of cardio-metabolic risk factors was considerable among military personnel.
- The findings suggest that implementing interventions for the control of cardio-metabolic risk factors among military personnel seems necessary.

## Background

The global prevalence of cardiovascular diseases and Metabolic syndrome (MetS) has increased over the last 20 years. The prevalence of Mets in men and women varies from 8% in India to 24% in USA, and from 7% in France to 43% in Iran, respectively [1]. Studies conducted on subjects over the past 20 years revealed that overweight, obesity, hypertension and hypercholesterolemia are the four leading causes of risk factors with the highest share of cardiovascular diseases [2, 3]. Mets is defined as a group of metabolic disorders that can lead to developing cardiovascular diseases, including central obesity, dyslipidemia, type II diabetes mellitus, certain cancers and all-cause mortality [1].

Sociodemographic factors (e.g. age, race and ethnicity), health behaviors (e.g. smoking, physical activity) and neuropsychiatric outcomes (depression, post-traumatic disorders) play a decisive role in the development of Mets [4–6]. Some of these factors are independently associated with military service [7, 8]. Military service personnel work in a unique environment characterized by high risk conditions and high levels of occupational stress [9]. It has been reported that military personnel with their heavy responsibilities are more likely to expose a greater risk of developing cardiovascular risk factors [10, 11].

Obesity and MetS have become the main health threat factors in military health system and their alarming incidence is a serious challenge for authorized organizations [12]. A study conducted on a population of military personnel in Iran reported that the prevalence of Mets, overweight and abdominal obesity in this group was estimated to be 11, 48 and 45%, respectively [13]. The prevalence of MetS in Chinese general population (16.5%) was much lower than that in the military population (35%) [14]. Obesity has been called as a serious national security threat by military institute in the United States [12]. A study on military personnel in Saudi Arabia revealed that the prevalence rates of overweight, obesity and current smoking were 41, 29 and 35% respectively [15].

There are numerous studies on the global prevalence of cardio metabolic risk factors (CMRFs) among military personnel. It is thus important to obtain an overall estimation on the prevalence of above-mentioned risk factors by synthesizing available studies. To date, the current study is the first meta-analysis conducted on this topic globally. Therefore, this study aimed to systematically review the literature on the estimation of the global pooled prevalence of CMRFs, including overweight, obesity, high low-density lipoprotein (LDL), high total cholesterol (TC), high triglyceride (TG), low high-density lipoprotein (HDL), hypertension (HTN) and high fasting blood sugar (FBS) in the military population.

## Methods

### Identification of relevant studies

This is a comprehensive systematic review of all available evidences on the prevalence of CMRFs in the military personnel. We developed a systematic review adhering to the PRISMA-P guidelines [16]. All the documents are based on the details of the study protocol. Registration number of current study in PROSPERO is CRD42018103345.

The main root of developing the search strategies is based on the two main components of “cardio metabolic risk factors” and “metabolic syndrome” in military personals. To assess the optimal sensitivity of search for documents, we simultaneously searched PubMed and NLM Gateway (for MEDLINE), Institute of Scientific Information (ISI), and SCOPUS as the main international electronic data sources (Additional file 1).

### Inclusion and exclusion criteria

All available observational studies conducted up to March 2018 c on relevant subjects were included. There was no limitation for the target groups in terms of age and gender and language of published studies. In situation of more than one paper from the one study, the most complete data were considered. We also excluded papers with duplicate citation. Non-peer reviewed articles, conference proceedings and book chapters were considered for more access to relevant data.

### Quality assessment and data extraction

After completing all three steps of data assessment for titles, abstracts and full texts, the full texts of each article selected were retrieved for more detailed analysis. The quality assessment and data extraction were followed a check list recorded citation, publication year, study year, place of study, type of study, population characteristics and methodological criteria (sample size, mean age, type of measure, results of measures and other information).

The whole process of searching for the data extraction and quality assessment was followed independently by two research experts. The kappa statistic for agreement of quality assessment was 0.94. Probable discrepancies between experts were resolved by discussion. Any disagreements were resolved by consensus by a third person. The quality assessment was performed using a validated quality assessment checklist for prevalence studies [17]. This tool comprises 10 items which covers methodological quality of prevalence studies, including sampling method (2 questions), data collection (5 questions) and data analysis (3 questions). Each item can be answered either Yes/No or Unclear/ Not applicable. The overall score for 10 studies was the total score ≥ 6, considered as acceptable in terms of quality.

### Statistical analysis

The prevalence and 95% confidence intervals (CI) were used for presenting the results. Chi-square based on Q test and I square statistics were used to assess the heterogeneity of reported prevalence among the studies. *P* < 0.05 was regarded as statistically significant at. Due to severe heterogeneity among studies regarding reported prevalence, the pooled prevalence was estimated using a random-effect meta-analysis proposed by Der-Simonian and Laird. We undertook a meta-regression analysis to assess the effect of study covariates, including the mean age of participants, quality score, type of personnel, and years of publication of reported prevalence. Meta-analysis was performed for risk factors reported in more than four studies. If a study was reported separately the prevalence of CMRFs over a time period, the weighted prevalence for the entire period would calculate and then this value could be considered as an overall prevalence in the meta-analysis. The prevalence of MetS was extracted according to International Diabetes Federation (IDF), World Health Organization (WHO) and National Cholesterol Education Program- Adult Treatment Panel III (ATPIII) criteria. Since most studies had reported MetS by ATP-III criteria, only these studies were included in meta-analysis. To assess the effect of each study on overall prevalence, we performed sensitivity analyses by sequentially removing each study and rerunning the analysis. Statistical analysis was performed using STATA software, V.11.1 (StataCorp LP, College Station, Texas, USA).

## Results

### Study selection process

**Fig. 1:**
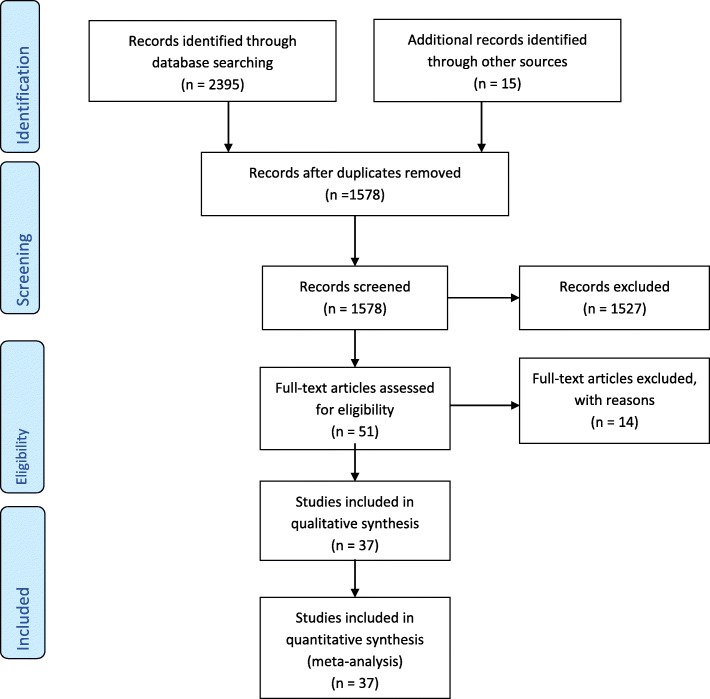
PRISMA 2009 flow diagram. *From:* Moher D, Liberati A, Tetzlaff J, Altman DG, The PRISMA Group (2009). *P*referred *R*eporting *I*tems for *S*ystematic Reviews and *M*eta-*A*nalyses: The PRISMA Statement. PLoS Med 6(7): e1000097. doi:10.1371/journal. pmed1000097. For more information, visit www.prisma-statement.org.

Figure 1 shows the flowchart of selection of studies for inclusion in the meta-analysis. In total, 2395 papers were identified after initial database search. Of these, 51 full-text papers were assessed for eligibility. In the next phase, 14 full text papers were excluded and finally 37 studies were eligible for inclusion in this meta-analysis: [9, 13, 15, 18–51].

### Study characteristics

The selected articles were published between 2001 and 2017. Out of 37 studies, 8 contained the prevalence information for navy, 16 for military personnel, 5 for army, 5 for soldier’s /warship personnel and 3 for air force staff. Six studies had reported trends in the prevalence of CMRFs over a time period [22, 24, 26, 28, 30, 40], so that their weighted prevalence was considered as an overall prevalence. Among all publications, 15 studies were conducted in the American countries [9, 19, 20, 24–27, 29–32, 36, 38, 41, 51], 13 in Europe [22, 28, 33–35, 37, 39, 40, 44, 45, 48–50] and 9 in Asia [13, 15, 18, 21, 23, 42, 43, 46, 47].

### Qualitative synthesis

Table 1 shows the general characteristics of the selected studies for the prevalence of MetS. According to ATPIII criteria, the highest and lowest prevalence rates of MetS were 39 and 9% in US mariners [31] and French military staffs [49], respectively. The prevalence range of MetS was 3.8–39% according to the different definition criteria.

**Table 1:** Characteristic of the selected studies on the prevalence of Mets

Author, year	Country	Study type	Study year	Study population	Sampling	Sample size	Mean age/ Range	Outcome	Definition/Criteria	Prevalence%(95% CI)
Payab, 2017 [13],	Iran	C/S	2015	Military	Convenience	2200	37.73	Mets	ATPIII	11.1 (9.8–12.5)
									ATPIII with waist> 90 cm	26.6 (24.7–28.5)
									ATPIII> 95 cm	19.6 (17.9–21.3)
Sharma, 2016 [18],	India	C/S	Not provided	Military aircrew	Convenience	210	20–50	Mets	MS-4	33.0 (26.6–39.7)
									ATPIII	11.9 (7.6–16.7)
									IDF	7.1 (4.0–11.7)
									WHO	3.8 (1.8–7.6)
Gasier, 2016 [20],	US	C	Not provided	Navy (Submariners)	Convenience	53	29	Mets	ATP-III	30.0 (18.7–44.5)
Baygi, 2016 [21],	Iran	C/S	2015	Seafarers	Convenience	234	36	Mets	IDF	14.9 (10.8–20.3)
Rhee, 2015 [23],	Korea	C/S	2014	Military aviators	Convenience	911	24–49	Mets	WHO	9.8 (7.9–11.9)
Herzog, 2015 [27],	US	C/S	2012	Military	Convenience	79,139	18–65	Mets	ATPIII	16.7 (15.7–16.2)
Filho, 2014 [9],	Brazil	C/S	2012	Military	Convenience	452	45.8	Mets	ATPIII	38.5 (34.0–43.2)
Scovill, 2012 [31],	US	C/S	Not provided	Mariner	Convenience	388	44	Mets	ATPIII	39.0 (34.1–43.9)
Hagnas, 2012 [33],	Finland	Prospectiv	Not provided	Military	Convenience	1046	19.2	Mets	IDF	6.1 (4.8–7.8)
Costa, 2011 [36],	Brazil	C/S	2008	Navy	Convenience	1383	30.7	Mets	IDF	17.6 (15.6–19.7)
Khazale, 2007 [43],	Jordan	C	2006	Air force	Convenience	111	32.5	Mets	ATPIII	18 (11.6–26.7)
Al-Qahtani, 2005 [47],	Saudi Arabia	C/S	2004	Soldiers	Convenience	1079	20–60	Mets	ATPIII	20.8 (18.4–23.3)
Athyros, 2005 [48],	Greece	C/S	2003	Military	Convenience	300	37.0	Mets	ATPIII	9.4 (6.4–13.3)
Bauduceau, 2005 [49],	France	C/S	2003	Military	Convenience	2045	38.6	Mets	ATPIII WHO	9.0 (7.8–10.3) 14.0 (12.5–15.6)

C/S: Cross-sectional; C: Cohort; Mets: Metabolic Syndrome; ATPIII: Adult Treatment Panel III; IDF: International Diabetes Federation; WHO: World Health Organization

Characteristics of the selected studies for the prevalence of overweight, generalized obesity and abdominal obesity are shown in Table 2. The highest prevalence of overweight (66%) and obesity (62%) was reported in Danish seafarers and the US submariners, respectively.

**Table 2:** Characteristic of the included studies on the prevalence of overweight, obesity and abdominal obesity

Author, year	Country	Study type	Study year	Study population	Sampling	Sample size	Mean age/ Range	Outcome	Definition/Criteria	Prevalence% (95% CI)
Payab, 2017 [13],	Iran	C/S	2015	Military	Convenience	2200	37.73	Overweight Obesity Abdominal Obesity	25.9 ≤ BMI < 29.9 kg/m^2^ BMI ≥ 30 kg/m^2^ WC > 90 cm	47.59 (45.4–49.7) 15.05 (13.6–16.6) 45.4 (43.3–47.5)
Rush, 2016 [19],	US	C/S	2001	Military	Randomly	77,047	42	Overweight Obesity	25 ≤ BMI < 29.9 kg/m^2^ BMI ≥ 30 kg/m^2^	51.0 (50.6–51.3) 23.0 (22.7–23.3)
Gasier, 2016 [20],	US	C	Not provided	Navy (Submariners)	Convenience	53	29	BF% Overweight Obesity	BF ≥ 25%	27.0 (15.7–40.6)
									25 ≤ BMI < 29.9 kg/m^2^	6.0 (1.5–16.6)
									BMI ≥ 30 kg/m^2^	62.0 (47.8–74.9)
Baygi, 2016 [21],	Iran	C/S	2015	Sefarers	Convenience	234	36	Abdominal obesity Excess weight	WC > 95 cm	38.5 (32.3–45.0)
									BMI > 25 kg/m^2^	51.1 (44.7–57.8)
Fajfrova,2016 [22],	Czech Republic	C/S		Armed Forces	Convenience	69,962	40	Overweight Obesity	–	51.5 (51.0–52.0) 14.0 (13.7–14.2)
Rhee, 2015 [23],	Korea	C/S	2014	Military aviators	Convenience	911	24–49	Abdominal obesity	WC > 90 cm	25.3 (22.5–28.2)
Reyes-Guzman, 2015 [24],	US	C/S	2008	Military	Randomly	90,905	25–46	Overweight Obesity	25 ≤ BMI < 29.9 kg/m^2^	47.8 (47.4–48.3)
									BMI ≥ 30 kg/m^2^	9.6 (9.4–9.7)
Lennon, 2015 [25],	US	C/S	2012	Sailor	Convenience	313,513	17–50	Obesity	BMI > 30 kg/m^2^	13.6 (13.4–13.7)
Hruby, 2015 [26],	US	C/S	2012	Army	Convenience	1,703,150	20–40	Overweight Obesity	25 ≤ BMI < 30 kg/m^2^ BMI ≥ 30 kg/m^2^	33.6 (33.5–33.6) 8.2 (8.1–8.2)
BinHoraib, 2013 [15],	Saudi Arabia	C/S	2009	Military	Multi-stage stratified random	10,229	34.1	Overweight Obesity Abdominal obesity	25 ≤ BMI < 30 kg/m^2^	40.9 (39.9–40.7)
									BMI ≥ 30 kg/m^2^	29.0 (28.1–29.9)
									WC > 90 cm	42.4 (41.4–43.3)
Binkowska-Bury, 2013 [28],	Poland	C/S	2010	Military	Convenience	37,916	19	Overweight Obesity	25 ≤ BMI < 29.9 kg/m^2^	12.6 (12.2–12.9)
									BMI ≥ 30 kg/m^2^	3.0 (2.8–3.1)
Marion,2012 [29],	US	C/S	2008	Navy	Convenience	26,341	26.5	Obesity	BMI ≥ 30 kg/m^2^	15.9 (15.4–16.3)
Smith, 2012 [30],	US	Not provided	2005	Military	Convenience	28,602	17–40	Excess weight	BMI ≥ 25 kg/m^2^	58.9 (58.3–59.4)
Scovill, 2012 [31],	US	C/S	Not provided	Mariner	Convenience	388	44	Obesity	BMI ≥ 30 kg/m^2^	61.0 (56.0–65.9)
Pasiakos, 2012 [32],	US	L	Not provided	Army	Convenience	209	21	Obesity	BMI ≥ 30 kg/m^2^	14.0 (9.6–19.5)
Sundin, 2011 [34],	UK	Not provided	2006	Armed Forces	Stratified Random Sampling	T:2470 M:2148 F:311	28.3	Overweight T M F Obesity T M F	25 ≤ BMI < 30 kg/m^2^	29.6 (27.7–31.4)
									BMI ≥ 30 kg/m^2^	30.5% (28.6–32.5) 27.1% (22.2–32.3) 13.5 (12.2–14.9) 13.5% (12.1–15.0) 13.5% (10.0–17.9)
Hansen, 2011 [35],	Denmark	Not provided	2010	Seafarers	Convenience	2101	18–64	Overweight	25 ≤ BMI < 30 kg/m^2^	66.0 (36.9–67.9)
Costa, 2011 [36],	Brazil	C/S	2008	Navy	Convenience	1383	30.7	Abdominal obesity	WC ≥ 90 cm	35.0 (32.5–37.6)
Mullie, 2010 [37],	Belgium	C/S	2007	Army	Random	974	44.0	Obesity	BMI ≥ 30 kg/m^2^	15.2 (13.3–17.9)
Wenzel, 2009 [38],	Brazil	C/S	2000	Military Air force	Convenience	380	19–49	Overweight Obesity	25 ≤ BMI < 30 kg/m^2^	36.0 (31.3–41.1)
									BMI ≥ 30 kg/m^2^	8.0 (5.5–11.2)
Saely, 2009 [39],	Switzerland	C	2004	Army	Convenience	56,784	19.7	Overweight Obesity	25 ≤ BMI < 30 kg/m^2^	16.8 (16.5–17.1)
									BMI ≥ 30 kg/m^2^	4.1 (3.9–4.2)
Mullie, 2008 [40],	Belgium	C/S	1992–2005	Army	Convenience	43,343	20–59	Overweight Obesity	25 ≤ BMI < 30 kg/m^2^ BMI ≥ 30 kg/m^2^	34.9 (34.4–35.3) 3.5 (3.3–3.6)
Napradit, 2007 [42],	Thailand	C/S	2005	Army	Convenience	4276	41.5	Overweight Obesity	25 ≤ BMI < 30 kg/m^2^ BMI ≥ 30 kg/m^2^	27.1 (25.7–28.4) 4.9 (4.3–5.6)
Khazale, 2007 [43],	Jordan	C	2006	Air force	Convenience	111	32.5	Abdominal obesity	WC > 102 cm	9.3 (4.6–16.3)
Hoeyer, 2005 [45],	Denmark	Not provided	Not provided	Seafarers	Convenience	1257	16–66	Overweight Obesity	25 ≤ BMI < 30 kg/m^2^	17.1 (15.1–19.2)
									BMI ≥ 30 kg/m^2^	5.8 (4.6–7.3)
Al-Qahtani, 2005 [46],	Saudi Arabia	C/S	2004	Soldiers	Convenience	1049	36.1	Overweight Obesity	25 ≤ BMI < 30 kg/m^2^	37.5 (34.5–40.4)
									BMI ≥ 30 kg/	31.6 (28.7–34.4)
Al-Qahtani, 2005 [47],	Saudi Arabia	C/S	2004	Soldiers	Convenience	1079	20–60	Abdominal Obesity	WC > 102 cm	33.1 (30.3–36.0)
Athyros, 2005 [48],	Greece	C/S	2003	Military	Convenience	300	37.0	Abdominal Obesity	WC > 102 cm	13.7 (10.1–18.2)
Bauduceau, 2005 [49],	France	C/S	2003	Military	Convenience	2045	38.6	Abdominal obesity	WC > 102 cm	17.0 (15.4–18.7)
Mazokopakis, 2004 [50],	Greece	C/S	1998	Warship personnel	Convenience	274	24.4	Overweight Obesity	25 ≤ BMI < 29.9 kg/m^2^	26.5 (21.2–31.9)
									BMI ≥ 30 kg/m^2^	4.7 (2.6–8.1)
Lindquist, 2001 [51],	US	C/S	1995–1998	Military	Convenience	33,457	20–35	Overweight	BMI ≥ 25 kg/m^2^	52.0 (51.4–52.5)

C/S: Cross-sectional; L: Longitudinal; BF: Body Fat; BMI: Body Mass Index; ATPIII: Adult Treatment Panel III; IDF: International Diabetes Federation; WC: Waist circumferences; F: Female; M: Male; T: Total

Table 3 shows the characteristics of the selected studies for the prevalence of abnormal lipid profile and other CMRFs. A study carried out by Smoley et al. [41] in the US found the highest prevalence (63%) of Pre-HTN. The highest and lowest prevalence rates of HTN were observed in the Brazilian military (55.8%) and the Iranian military (2.6%), respectively. The highest and lowest prevalence rates of high TG were 50.9% [9] and 5.0% [32] for American military personnel.

**Table 3:** Characteristic of the included studies on the prevalence of high level lipid profile, high glycemic indices and hypertension

Author, year	Country	Study type	Study year	Study population	Sampling	Sample size	Mean age/ Range	Outcome	Definition/Criteria	Prevalence% (95% CI)
Payab, 2017 [13],	Iran	C/S	2015	Military	Convenience	2200	37.73	HTN	SBP ≥130 mmHg or DBP ≥85 mmHg	2.6 (1.98–3.37)
Gasier, 2016 [20],	US	C	Not provided	Obese Navy (Submariners)	Convenience	53	29	Insulin resistant	HOMA> 2.73	30.0 (18.7–44.5)
Baygi, 2016 [21],	Iran	C/S	2015	Sefarers	Convenience	234	36	High TG	TG ≥150 mg/dl	25.2 (20.3–31.8) 26.5 (21.1–32.7) 26.5 (21.1–32.7) 28.2 (22.6–34.5) 19.2 (14.5–25.0) 23.1 (17.9–29.11)
								Low HDL	HDL < 40 mg/dl	
								High LDL	LDL.130 mg/dl	
								High TC	TC ≥ 200 mg/dl	
								HTN	SBP ≥130 mmHg or DBP ≥85 mmHg	
								High FBS	FBS > 100 mg/dl	
Rhee, 2015 [23],	Korea	C/S	2014	Military aviators	Convenience	911	24–49	High BP Impaired glucose High TG Low HDL	SBP ≥130 mmHg or DBP ≥85 mmHg FBS ≥ 100 mg/dl TG ≥150 mg/dl HDL < 40 mg/dl	31.7 (28.7–34.9) 19.0 (16.5–21.7) 16.6 (14.2–19.1) 7.9 (6.3–9.9)
Filho, 2014 [9],	Brazil	C/S	2012	Military	Convenience	452	45.8	HTN	SBP ≥130 mmHg or	55.8 (51.0–60.4) 50.9 (46.2–55.6) 30.5 (26.4–35.0) 30.5 (26.4–35.0)
								High TG	DBP ≥85 mmHgTG	
								Low HDL	≥150 mg/dl	
								High FBS	HDL < 40 mg/dl FBS > 100 mg/dl	
Scovill, 2012 [31],	US	C/S	Not provided	Mariner	Convenience	388	44	HTN	SBP ≥130 mmHg or	42.0 (37.1–47.1) 42.0 (37.1–47.1) 47.0 (41.8–52.0) 22.0 (17.9–26.4)
								High TG	DBP ≥85 mmHg	
								Low HDL	TG ≥150 mg/dl	
								High FBS	HDL < 40 mg/dl LDL > 130 mg/dlFBS ≥ 100 mg/dl	
Pasiakos, 2012 [32],	US	L	Not provided	Army	Convenience	209	21	High TC High TG Low HDL High LDL High FBS	TC ≥ 200 mg/dl TG ≥150 mg/dl HDL < 40 mg/dl LDL > 130 mg/dl FBS > 100 mg/dl	8.0 (4.9–12.9) 5.0 (2.4–8.9) 33.0 (26.8–39.9) 39.0 (32.2–45.7) 8.0 (4.9–12.9)
Costa, 2011 [36],	Brazil	C/S	2008	Navy	Convenience	1383	30.7	Low HDL HTN High TG High FBS	HDL < 40 mg/dl SBP ≥130 mmHg or DBP ≥85 mmHg TG ≥150 mg/dl FBS ≥ 100 mg/dl	43.0 (40.4–45.7) 26.3 (24.0–28.7) 19.3 (17.3–21.5) 6.6 (5.4–8.0)
Mullie, 2010 [37],	Belgium	C/S	2007	Army	Random	974	44.0	High TC	TC ≥ 190 mg/dl	65.0 (61.7–67.9)
Wenzel, 2009 [38],	Brazil	C/S	2000	Military Air force	Convenience	380	19–49	HTN	SBP ≥140 mmHg or DBP ≥90 mmHg	22.0 (18.1–26.7)
Saely, 2009 [39],	Switzerland	C	2004	Army	Convenience	56,784	19.7	Pre-HTN HTN High TC	120 ≤ SBP < 139 mmHg SBP ≥140 mmHg or DBP ≥90 mmHg TC ≥ 190 mg/dl	61.4 (61.0–61.8) 26.8 (26.4–27.2) 7.8 (7.6–8.0)
Smoley, 2008 [41],	US	C/S	2004	Service members	Convenience	15,391	27.8	Pre HTN HTN	120 ≤ SBP < 139 mmHg or 80 ≤ DBP < 89 mmHg SBP ≥140 mmHg or DBP ≥90 mmHg	63.0 (62.2–63.7) 11.0 (105–11.5)
Napradit, 2007 [42],	Thailand	C/S	2005	Army	Convenience	4276	41.5	HTN	SBP ≥140 mmHg or DBP ≥90 mmHg	34.5 (33.1–35.9)
Khazale, 2007 [43],	Jordan	C	2006	Air force	Convenience	111	32.5	High SBP High DBP High TC Low HDL High FBS	SBP > 130 mmHg DBP > 85 mmHg TC ≥ 150 mg/dl HDL < 40 mg/dl FBS > 100 mg/dl	9.6 (4.6–16.3) 23.1 (13.8–29.6) 52.2 (42.6–61.7) 38.7 (29.7–48.5) 9.6 (4.6–16.3)
Vaicaitiene, 2006 [44],	Lithuania	C/S	Not provided	Military	Random	200	25–54	High TC	TC ≥ 240 mg/dl	43.4 (36.5–50.6)
Al-Qahtani, 2005 [47],	Saudi Arabia	C/S	2004	Soldiers	Convenience	1079	20–60	High TG High BP	TG ≥150 mg/dl SBP > 130 mmHg DBP > 85 mmHg	32.2 (29.4–35.5) 29.5 (26.8–32.3)
Athyros, 2005 [48],	Greece	C/S	2003	Military	Convenience	300	37.0	High FBS High TG Low HDL Impaired Glucose	FBS > 100 mg/dl TG ≥150 mg/dl HDL < 40 mg/dl FBS > 100 mg/dl	4.0 (2.2–7.1) 25.0 (20.3–30.4) 9.4 (6.4–13.3) 3.0 (1.5–5.8) 1.0 (0.3–3.1)
Bauduceau, 2005 [49],	France	C/S	2003	Military	Convenience	2045	38.6	HTN High TG Low HDL High FBS	SBP > 130 mmHg or DBP > 85 mmHg TG ≥150 mg/dl HDL < 40 mg/dl FBS > 100 mg/dl	51.0 (48.7–53.1) 17.0 (15.4–18.7) 9.6 (8.4–10.9) 5.0 (4.1–6.0)

C/S: Cross-sectional; C: Cohort; L: Longitudinal; ATPIII: Adult Treatment Panel III; IDF: International Diabetes Federation; WHO: World Health Organization; FBS, fasting blood sugar; TC, total cholesterol; TG, triglycerides; LDL-C, low-density lipoprotein cholesterol; HDL-C, high-density lipoprotein cholesterol; BP, blood pressure; SBP: Systolic blood pressure; DBP: Diastolic blood pressure; HTN: Hypertension; HOMA: Homeostasis model assessment

### Meta-analysis

The results of meta-analysis are shown in Table 4. The total sample size of the studies included in meta-analysis was *n* = 12,153,936. The study population consisted of men and women aged 16–66 years. The eligible studies for estimation of the prevalence of MetS, overweight, obesity, high LDL, high TC and HTN were 10, 19, 22, 29, 6 and 13, respectively.

**Table 4:** The pooled prevalence of cardiometabolic risk factors in Military Population at global level using random effect meta-analysis method

Variables	No. of studies	Sample Size	Prevalence (CI 95%)	Model	I^2^(%)	*P-value
MetS	10	4,912,369	21 (17–25)	Random	97	< 0.001
Overweight	19	2,867,867	35 (31–39)	Random	99	< 0.001
Obesity	22	3,211,654	14 (13–16)	Random	99	< 0.001
Abdominal obesity	8	17,581	29 (20–39)	Random	99	< 0.001
HTN	13	816,414	26 (19–34)	Random	99	< 0.001
High TG	9	7001	24 (16–31)	Random	98	< 0.001
Low HDL	9	6033	28 (17–38)	Random	99	< 0.001
High LDL	29	157,730	32 (27–36)	Random	99	< 0.001
High TC	6	58,512	34 (10–57)	Random	99	< 0.001
High FBS	6	4436	9 (5–12)	Random	92	< 0.001

*According to Q test (Chi-square test)

**Fig. 2:**
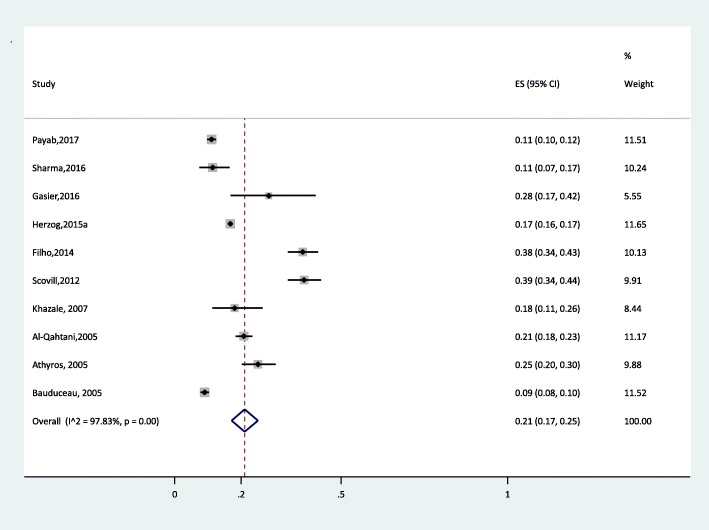
Forest plot of MetS global prevalence using random-effect model

According to random effect meta-analysis, the rates of the global pooled prevalence (95% confidence interval) of MetS, high LDL, high TC, high TG, low HDL and high FBS were 21% (17–25), 32% (27–36), 34% (10–57), 24% (16–31), 28% (17–38) and 9% (5–12), respectively. Moreover, the rates of the global estimated pooled prevalence of overweight, generalized obesity, abdominal obesity and HTN were 35% (31–39), 14% (13–16), 29% (20–39) and 26% (19–34), respectively. Figure 2 shows a forest plot of eligible articles for the estimation of MetS prevalence.

### Quality assessment

The quality assessment of the included studies was performed by using a critical appraisal tool for use in systematic reviews addressing questions of prevalence. Accordingly, all studies had an acceptable quality score (Table 5).

**Table 5:** Quality assessment of the included studies

Study	Total score	Item _1_	Item _2_	Item _3_	Item _4_	Item _5_	Item _6_	Item _7_	Item _8_	Item _9_	Item _10_
Payab, 2017	7	N	Y	Y	Y	N	Y	Y	N	Y	Y
Sharma, 2016	5	N	–	Y	Y	N	Y	N	N	Y	Y
Rush, 2016	6	N	Y	Y	Y	N	Y	N	N	Y	Y
Gasier, 2016	3	N	N	N	N	N	Y	UC	N	Y	Y
Baygi, 2016	7	N	Y	Y	Y	NA	Y	Y	N	Y	Y
Fajfrova,2016	4	N	Y	Y	Y	NA	N	Y	N	N	N
Rhee, 2015	8	N	Y	Y	Y	NA	Y	Y	Y	Y	Y
Reyes-Guzman, 2015	7	N	Y	Y	Y	N	Y	N	Y	Y	Y
Lennon, 2015	6	N	Y	Y	Y	NA	Y	N	N	Y	Y
Hruby, 2015	7	N	Y	Y	Y	NA	Y	UC	Y	Y	Y
Herzog, 2015	7	N	Y	Y	Y	NA	Y	UC	Y	Y	Y
Filho, 2014	5	N	N	Y	Y	N	Y	UC	N	Y	Y
BinHoraib, 2013	8	N	Y	Y	Y	N	Y	Y	Y	Y	Y
Binkowska-Bury, 2013	4	N	Y	Y	N	NA	Y	UC	Y	N	N
Marion,2012	7	N	Y	Y	Y	NA	Y	UC	Y	Y	Y
Smith, 2012	7	N	Y	Y	Y	NA	Y	UC	Y	Y	Y
Scovill, 2012	3	N	Y	Y	N	N	Y	UC	N	N	N
Pasiakos, 2012	5	N	N	Y	Y	N	Y	UC	Y	N	Y
Hagnas, 2012	3	N	Y	Y	N	N	N	Y	N	N	N
Sundin, 2011	7	N	Y	Y	Y	N	Y	N	Y	Y	Y
Hansen, 2011	7	N	Y	Y	Y	NA	Y	Y	N	Y	Y
Costa, 2011	6	N	N	Y	Y	N	Y	N	Y	Y	Y
Mullie, 2010	6	N	N	Y	Y	Y	Y	UC	N	Y	Y
Wenzel, 2009	7	N	N	Y	Y	N	Y	Y	Y	Y	Y
Saely, 2009	5	N	Y	Y	N	NA	Y	UC	N	Y	Y
Mullie, 2008	7	N	Y	Y	Y	N	Y	N	Y	Y	Y
Smoley, 2008	8	N	Y	Y	Y	NA	Y	Y	Y	Y	Y
Napradit, 2007	7	N	Y	Y	Y	N	Y	N	Y	Y	Y
Khazale, 2007	5	N	Y	N	Y	N	Y	N	N	Y	Y
Vaicaitiene, 2006	7	N	Y	Y	Y	N	Y	Y	N	Y	Y
Hoeyer, 2005	5	N	N	Y	Y	N	Y	N	N	Y	Y
Al-Qahtani, 2005	6	N	N	Y	N	Y	Y	N	Y	Y	Y
Al-Qahtani, 2005	6	N	N	Y	N	Y	Y	N	Y	Y	Y
Athyros, 2005	6	N	Y	Y	Y	N	Y	N	N	Y	Y
Bauduceau, 2005	5	N	Y	Y	Y	N	Y	Y	N	N	N
Mazokopakis, 2004	3	N	N	Y	Y	N	Y	N	N	N	N
Lindquist, 2001	6	N	Y	Y	Y	Y	Y	N	Y	N	N

Item _1_: Was the sample representative of the target population?

Item _2_: Were study participants recruited an appropriate way?

Item _3_: Was the sample size adequate?

Item _4_: Where the study subjects and setting described in detail?

Item _5_: Was the data analysis conducted with sufficient coverage of the identified sample?

Item _6_: Were objective, standard criteria used for measurement of the condition?

Item _7_: Was the condition measured reliably?

Item _8_: Was there appropriate statistical analysis?

Item _9_: Are all important confounding factors/subgroups/different identified and accounted for?

Item _10_: Were subpopulations identified using objective criteria?

**Y**: Yes, **N**: No, **UC**: Unclear, **NA**: Not applicable

### Meta-regression

Results of meta-regression analysis demonstrated that effect of study characteristics, including the mean age of participant, quality score, type of personnel, and years of publication on reported prevalence was not statistically significant (*p* > 0.05).

### Sensitivity analysis

Sensitivity analyses were performed to assess effect of each individual study on pooled prevalence rates. The results showed that no significant changes in in the pooled prevalence was found in the included studies (p > 0.05).

## Discussion

To the best of our knowledge, this is the first meta-analysis to estimate the global pooled prevalence of CMRFs in the military population. In the current study, the overall prevalence of MetS was estimated to be 21% according to ATP-III criteria. The prevalence of Mets was among Iranian male military personnel 11% [13]. Corresponding prevalence was 35% in Chinese military population, while it was 17% in the Chinese general population [14]. The prevalence of Mets was 39% among Brazilian soldiers [9], whereas it was 15% among Royal Jordanian Air Force pilots [4]. In a study conducted by Baygi et al. on Iranian seafarers demonstrated that the prevalence of Mets was 15% which was lower than that (33%) for urban dwellers of Tehran [21]. The wide variation in these prevalence rates may be due to differences in study samples, age and gender.

In the present study, the estimated prevalence rates of overweight, obesity and abdominal obesity were 35, 14 and 29%, respectively. Bin Horaib et al. in their study of 5 military regions of Kingdom of Saudi Arabia among 10,500 active military personnel reported that the proportions of overweight, obesity and abdominal obesity were 41, 29 and 42%, respectively [15]. The prevalence rate of overweight was 52% in the U.S. navy [51], whereas it was 66% among Danish seafarers [35]. Using the dissimilar cutoff points and including females in some of the studies may explain differences between the prevalence figures. Because of the nature of their job, military individuals are generally assumed to be healthier. However, our findings showed an alarming trend in the global prevalence rates of overweight and obesity, which might be due to unhealthy diet practice among military personnel [13].

In the present study, the reported prevalence rates of Pre-HTN and HTN were 62 and 26%, respectively. A study conducted on male subjects in Saudi Arabia showed that the prevalence rate of HTN was 33%, indicating a progressive increase in body fat with age [52]. The results of a National survey conducted in the U.S. demonstrated that the estimated age-adjusted prevalence of HTN was 27% in men and 30% in women [53]. The corresponding estimate in general population of Korea was 33%, increased progressively with age from 14% among 14–24-year-olds to 71% among subjects aged 75 years or older [54]. The prevalence rate of HTN in people with regular and intensive physical activity was 13% lower than that in their non-active peers [55]. Our results showed that the prevalence rate of HTN in military personnel was 26% that was lower than that in the general population. This is likely explained by a reverse association between intensive physical activity and HTN.

Based on our findings, the estimated prevalence rates of high TG, low HDL, high LDL and high TC were 24, 28, 32 and 34%, respectively. The results of a study conducted among 911 Korean military aviators demonstrated that the prevalence rates of elevated TG and reduced HDL were 16.6 and 7.9%, respectively [23]. The prevalence rates of mentioned figures in the general Korean population were significantly lower than those of their peers in Air Force [56]. A meta-analysis conducted by Tabatabaei et al. in Iranian general population showed that these figures for high TG, low HDL, high LDL and high TC were 41.6, 46, 35.5 and 43.9%, respectively [57]. The significant differences between general population and military personnel with respect to lipid profile could be explained by their strict standards for physical activity on a regular basis as which might have positive effects on their overall health status.

In the current study, the overall prevalence rates of high FBS and diabetes were 9 and 5%, respectively. The global prevalence rare of diabetes for all age groups has been estimated to be 2.8% in 2000 and 4.4% in 2030 [58]. The results of a study performed in Greece showed that the prevalence rate of diabetes was 10.6% in general population and 3.0% among military staff [48]. This is likely due to higher physical activity levels in the military personnel compared to their peers in the general population. Additionally, nutrition and physical activity of military individuals are strictly controlled for maintaining their healthy body weight which has a positive effect on managing FBS level and preventing Diabetes and other non-communicable diseases and their risk factors.

The limitations of this study are as follows, in most of the included studies, convenience sampling was used to estimate the prevalence which might be decreased generalizibiability of reported prevalence. Moreover, definition of some cardio- metabolic risk factors in the included primary studies was heterogeneous which the pooled prevalence might be limited by the different definitions.

## Conclusions

The overall estimated prevalence of some cardio-metabolic risk factors was estimated to be higher in military personnel. Therefore, this study provides strong evidence to the military healthcare providers’ and policy makers for devising and implementing feasible interventions in order to control risk factors in this occupation. Moreover, further studies are needed to identify associated risk factors and reveal best predictors of high-risk subpopulation.

## Supplementary information

**Additional file 1.** Search strategy.

## Data Availability

Data sharing is not applicable to this article as no datasets were generated or analyzed during the current study.

## Abbreviations

ATPIII: National Cholesterol Education Program- Adult Treatment Panel III.
CI: Confidence Intervals
CMRFs: Cardiometabolic Risk Factors
FBS: Fasting Blood Sugar
HDL: High-Density Lipoprotein
HTN: Hypertension
IDF: International Diabetes Federation
ISI: Institute of Scientific Information
LDL: Low-Density Lipoprotein
MetS: Metabolic Syndrome
TC: Total Cholesterol
TG: Triglyceride
WHO: World Health Organization
