# Supplementary material for: Global prevalence of cardiometabolic risk factors in the military population: a systematic review and meta-analysis
Source: BMC Endocr Disord. 2020 Jan 13;20:8. doi: 10.1186/s12902-020-0489-6 (PMC6958577; doi:10.1186/s12902-020-0489-6)
Supplement: Supplementary file 1 — Additional file 1. Search strategy. [file 12902_2020_489_MOESM1_ESM.docx]

**Appendix 1: Search strategy**

| PubMed |
| --- |
| ( (("X Syndrome"[Title/Abstract] OR "Syndrome X"[Title/Abstract]) OR "Insulin Resistance"[Title/Abstract]) OR "Cardiovascular Syndrome"[Title/Abstract]) OR Diabetes[Title/Abstract] OR "HDL"[Title/Abstract] OR "LDL"[Title/Abstract] OR "TG"[Title/Abstract] OR "hyper lipid"[Title/Abstract] OR "hyper cholesterol"[Title/Abstract] OR cardiometabolic[Title/Abstract] OR cardio-metabolic[Title/Abstract] OR "cardio metabolic"[Title/Abstract] OR "Metabolic Syndrome X"[Mesh] OR "Obesity, Abdominal"[Mesh] OR "Insulin Resistance"[Mesh] OR "Obesity"[Mesh] OR "Diabetes Mellitus"[Mesh] OR "Cholesterol, LDL"[Mesh] OR "Cholesterol, HDL"[Mesh] OR "Stroke"[Mesh]) OR "Heart Failure"[Mesh]) OR "Myocardial Infarction"[Mesh]) OR "Cardiovascular Diseases"[Mesh])) AND (("seafarer "[Mesh] OR " Sailor"[Title/Abstract] OR navigator [Title/Abstract] OR mariner [Title/Abstract] OR seagoing))) |
| Scopus |
| (((TITLE-ABS-KEY ( "Syndrome Metabolic" ) )  OR  ( ( TITLE-ABS-KEY ( "Metabolic Syndrome " )  OR  TITLE-ABS-KEY ( "Syndrome X ') OR TITLE-ABS-KEY("  x  AND syndrome  ") OR TITLE-ABS-KEY("  "Insulin Resistance " ) ) ) )  OR  ( ( ( TITLE-ABS-KEY ( "Cardiovascular Syndrome " )  OR  TITLE-ABS-KEY ( diabetes )  OR  TITLE-ABS-KEY ( "FPG" )  OR  TITLE-ABS-KEY ( "Fasting Plasma Glucose " ) ) )  OR  ( ( TITLE-ABS-KEY ( "HDL" )  OR TITLE-ABS-KEY ( "LDL" )  OR  TITLE-ABS-KEY ( "hyper lipid" )  OR  TITLE-ABS-KEY ( "hyper cholesterol" )  OR  TITLE-ABS-KEY ( cardiometabolic )  OR  TITLE-ABS-KEY ( cardio-metabolic )  OR  TITLE-ABS-KEY ( "cardio metabolic" )  OR  TITLE-ABS-KEY ( obesity ) OR  TITLE-ABS-KEY ( " Insulin Resistance " ) R  TITLE-ABS-KEY ( " Cholesterol " ) OR  TITLE-ABS-KEY ( "Stroke" ) OR  TITLE-ABS-KEY ( "Myocardial Infarction" ) OR  TITLE-ABS-KEY ( "Heart Failure" ) OR  TITLE-ABS-KEY ( "Cardiovascular Diseases" ) )  AND  ( TITLE-ABS-KEY )) " seafarer " OR  ( ( TITLE-ABS-KEY ( " navigator " )    OR  ( ( TITLE-ABS-KEY ( " mariner " )   OR  ( ( TITLE-ABS-KEY ( " Sailor " ) )) |
| ISI/WOS |
| TOPIC: ("Syndrome Metabolic") ORTOPIC: ("Metabolic Syndrome ") ORTOPIC: ("Syndrome X") ORTOPIC: ("X Syndrome") ORTOPIC: ("Insulin Resistance") ORTOPIC: ("Cardiovascular Syndrome ") ORTOPIC: (diabetes) ORTOPIC: ("FPG") ORTOPIC: ("Fasting Plasma Glucose") ORTOPIC: ("HDL") ORTOPIC: ("LDL") ORTOPIC: ("hyper lipid") ORTOPIC: ("hyper cholesterol") ORTOPIC: (cardiometabolic) ORTOPIC: (cardio-metabolic) ORTOPIC: ("cardio metabolic") ORTOPIC: ("Myocardial Infarction ")ORTOPIC: ("Stroke ")  ORTOPIC: ("Heart Failure ")ORTOPIC: ("Cardiovascular Diseases ") Indexes=SCI-EXPANDED, SSCI, CPCI-S, CPCI-SSH, ESCI Timespan=All yearsTOPIC: ) seafarer (ORTOPIC: ("navigator ") ORTOPIC: ("mariner ") ORTOPIC: (Sailor)Timespan=All years ANDIndexes=SCI-EXPANDED, SSCI, CPCI-S, CPCI-SSH Timespan=All years |
